# Synthetic lethality between PAXX and XLF in mammalian development

**DOI:** 10.1101/gad.290510.116

**Published:** 2016-10-01

**Authors:** Gabriel Balmus, Ana C. Barros, Paul W.G. Wijnhoven, Chloé Lescale, Hélène Lenden Hasse, Katharina Boroviak, Carlos le Sage, Brendan Doe, Anneliese O. Speak, Antonella Galli, Matt Jacobsen, Ludovic Deriano, David J. Adams, Andrew N. Blackford, Stephen P. Jackson

**Affiliations:** 1 Wellcome Trust/Cancer Research UK Gurdon Institute, University of Cambridge, Cambridge CB2 1QN, United Kingdom;; 2 Wellcome Trust Sanger Institute, Cambridge CB10 1HH, United Kingdom;; 3 Department of Biochemistry, University of Cambridge, Cambridge CB2 1GA, United Kingdom;; 4 Department of Immunology, University of Cambridge, Cambridge CB2 1GA, United Kingdom;; 5 Department of Genomes and Genetics, Institut Pasteur, 75015 Paris, France;; 6 AstraZeneca, Cambridge CB4 0FZ, United Kingdom;; 7 Weatherall Institute of Molecular Medicine, University of Oxford, John Radcliffe Hospital, Oxford OX3 9DS, United Kingdom;; 8 Cancer Research UK/Medical Research Council Oxford Institute for Radiation Oncology, Department of Oncology, University of Oxford, Oxford OX3 7DQ, United Kingdom

**Keywords:** ATM, development, NHEJ, PAXX, synthetic lethality, XLF

## Abstract

PAXX was identified recently as a novel nonhomologous end-joining DNA repair factor in human cells. To characterize its physiological roles, we generated *Paxx*-deficient mice. Like *Xlf*^−/−^ mice, *Paxx*^−/−^ mice are viable, grow normally, and are fertile but show mild radiosensitivity. Strikingly, while *Paxx* loss is epistatic with *Ku80*, *Lig4*, and *Atm* deficiency, *Paxx/Xlf* double-knockout mice display embryonic lethality associated with genomic instability, cell death in the central nervous system, and an almost complete block in lymphogenesis, phenotypes that closely resemble those of *Xrcc4*^−/−^ and *Lig4*^−/−^ mice. Thus, combined loss of *Paxx* and *Xlf* is synthetic-lethal in mammals.

## Abstract (precis)

Balmus et al. report that Paxx/Xlf double-knockout mice display embryonic lethality associated with genomic instability, cell death in the central nervous system, and an almost complete block in lymphogenesis, phenotypes that closely resemble those of Xrcc4^−/−^ and Lig4^−/−^ mice.

DNA double-strand breaks (DSBs) are extremely toxic lesions that must be repaired for an organism to pass on its genetic material intact to the next generation (Jackson and Bartek 2009). Cells have evolved two principal DSB repair pathways to address this challenge: homologous recombination (HR) and nonhomologous end-joining (NHEJ). HR is restricted to S and G2 phases of the cell cycle because it requires a sister chromatid as the template for repair. In contrast, NHEJ is the dominant DSB repair pathway throughout interphase in mammalian cells, although it is restrained during DNA replication (Beucher et al. 2009; Karanam et al. 2012). NHEJ is initiated by Ku, a ring-shaped heterodimeric protein complex consisting of Ku70 and Ku80 subunits that specifically recognizes DSB ends (Grundy et al. 2014). Ku forms a platform for the downstream recruitment of core NHEJ factors such as DNA ligase IV (LIG4); its stable binding partner, XRCC4; and XLF, a protein structurally related to XRCC4 (Ochi et al. 2014).

Most DSBs in vertebrate cells are generated by agents such as ionizing radiation (IR) or molecules that directly damage DNA by chemically reacting with it or through processing of other DNA lesions during DNA replication or mitosis. Additionally, some DSBs are induced deliberately by enzymatic cleavage in certain cell types at various stages of development; for example, to generate immune receptor diversity in B and T lymphocytes during variable, diversity, and joining [V(D)J] recombination (Jackson and Bartek 2009). NHEJ plays a central role in V(D)J recombination, a fact highlighted by the severe immunodeficiency found in some human patients and mice with NHEJ defects (Woodbine et al. 2014).

Mouse models of NHEJ deficiency show both overlapping and unique features. Ku-deficient mice are subviable and fertile but have profound growth defects, increased neuronal cell death, and immunodeficiency (Nussenzweig et al. 1996; Zhu et al. 1996; Ouyang et al. 1997; Gu et al. 2000). Loss of *Xrcc4* or *Lig4* results in a more severe phenotype with embryonic growth defects, blocked lymphogenesis, and late embryonic lethality associated with a large increase in cell death in the developing central nervous system (CNS) (Barnes et al. 1998; Frank et al. 1998; Gao et al. 1998). In contrast, *Xlf*^−/−^ mice have a relatively mild phenotype with no growth defects, neuronal cell death, or overt immunodeficiency despite XLF being a core NHEJ factor (Ahnesorg et al. 2006; Buck et al. 2006; Li et al. 2008). Furthermore, *Xlf*^−/−^ B cells perform V(D)J recombination at almost wild-type levels, which explains the lack of significant immunodeficiency in these mice and suggests that compensatory mechanisms can mitigate loss of XLF in developing lymphocytes. One of these mechanisms comprises the ATM–H2AX–53BP1 axis of DSB repair, as combined loss of any one of these factors with XLF deficiency causes profound defects in V(D)J recombination and lymphocyte development even though loss of any of these proteins individually is not significantly detrimental to these processes (Zha et al. 2011; Liu et al. 2012; Oksenych et al. 2012).

Recently, we and others identified a third XRCC4-like NHEJ protein, called PAXX (Craxton et al. 2015; Ochi et al. 2015; Xing et al. 2015). PAXX is required for cellular resistance to DSB-inducing agents and is rapidly recruited to DNA damage sites, where it stabilizes NHEJ factors on chromatin and promotes DNA repair by NHEJ. In vitro, PAXX can stimulate DNA end ligation in the presence of LIG4, XRCC4, and Ku, with most if not all of these functions requiring its direct binding to Ku. However, it is still unknown whether PAXX functions similarly to other core NHEJ factors in physiological settings or how PAXX loss might impact at the organism level in terms of growth and development.

In this study, we describe the generation and characterization of PAXX-deficient mice. Like *Xlf*^−/−^ mice, *Paxx*^−/−^ mice are viable, grow normally, and are fertile. However, these mice are radiosensitive and show a mild reduction in splenic lymphocyte numbers. Strikingly, combined loss of *Paxx* and *Xlf* is synthetic-lethal, as the majority of double-knockout embryos dies before birth with significant growth defects, increased genomic instability and subsequent cell death in the developing CNS, and an almost complete block in lymphocyte development, phenotypes that are strongly reminiscent of *Xrcc4*^−/−^ or *Lig4*^−/−^ mice. Thus, PAXX and XLF share a redundant function that is critical for DNA repair during mammalian development.

## Results and Discussion

### Generation of Paxx^−/−^ mice

**Figure 1.**
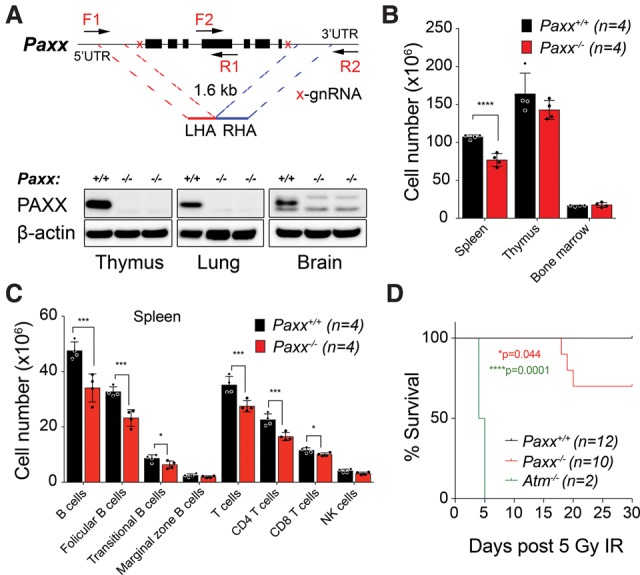
*Paxx*^−/−^ mice are viable and show increased sensitivity to IR. (*A*) Schematic representation of the CRISPR design deployed to delete the entire coding sequence of the *Paxx* locus on mouse chromosome 2. Representative Western blot showing PAXX protein level in wild-type versus *Paxx* knockout thymi, lungs, and brains. β-Actin was the loading control. (*B*) *Paxx*^−/−^ mice show a significant reduction of total cell numbers in the spleen ([****] *P* < 0.0001, Student's *t* test; 6-wk-old female mice; mean ± SD) but not in the thymus or bone marrow when compared with *Paxx*^+/+^ mice. (*C*) B-cell and T-cell distribution in the spleens of *Paxx*^+/+^ as compared with *Paxx*^−/−^ littermate mice. (***) *P* < 0.001; (*) *P* < 0.05, Student's *t* test. Mean ± SD. Leukocytes were identified as CD45^+^. B cells were defined as CD19^+^ with the subsets on the basis of CD21 and CD23 expression. (Follicular B) CD23 + CD21^low/neg^; (marginal zone B) CD23^low/neg^ CD21^high^; (transitional B) CD23^low^ CD21^low^. NK cells were defined as NK1.1^+^ CD3^−^, and T cells were defined as CD3^+^ NK1.1^−^ with T-cell subsets on the basis of surface CD4 or CD8 expression. (*D*) Mice (12–14 wk old) of the indicated genotypes were exposed to 5 Gy of whole-body IR. Kaplan-Meyer χ^2^ statistical analysis showed no lethality in *Paxx*^+/+^ mice but increased sensitivity to IR in *Paxx*^−/−^ mice. *Atm*^−/−^ mice were used as positive controls.

To characterize PAXX function in mice, we disrupted the *Paxx* locus in C57BL/6NTac zygotes using CRISPR–Cas9 (Wright et al. 2016) by injection of wild-type Cas9 mRNA together with two small guide RNAs (sgRNAs) and an oligonucleotide template to collapse the entire *Paxx* genomic region, resulting in a 1.6-kb deletion (Fig. 1A). For ease of mutant selection when using Cas9, *Paxx* sgRNAs were coinjected with an sgRNA to disrupt exon 1 of the tyrosinase gene (*Tyr*) to yield an albino phenotype in successfully targeted mice (Supplemental Fig. S1A,B). We identified four *Paxx* F0 founders, all of which showed the albino phenotype (Supplemental Fig. S2A,B). Upon confirmation of *Paxx* locus deletion by PCR and sequencing (Supplemental Fig. S2C), we backcrossed founders to the C57BL/6NTac original background for three generations to segregate the *Tyr* deletion and any off-target mutations. Resulting mice showed complete absence of PAXX protein in tissues such as thymus, lung, and brain that normally highly express *Paxx* (Fig. 1A).

*Paxx*^−/−^ mice were born at the expected Mendelian frequencies, were fertile, and displayed no developmental or hematological abnormalities (Supplemental Fig. S2D,E; Supplemental Table S1). *Paxx*^−/−^ mice also showed no increased tumor predisposition up to 400 d (data not shown) and no significant increase in micronucleus formation (Supplemental Fig. S3A), a sensitive method for detecting genomic instability in vivo (Balmus et al. 2015).

### Paxx^−/−^ mice are radiosensitive but show no overt immune phenotype

In light of the immune defects of NHEJ-deficient mouse models, we examined whether *Paxx*^−/−^ mice displayed any overt immunodeficiency by analyzing leukocyte numbers. We detected a modest reduction in cell counts in the spleens of *Paxx*^−/−^ mice compared with wild-type littermates, but this was not observed in the thymus or bone marrow (Fig. 1B). Splenic cell reduction was due to decreased B-cell and T-cell populations, while the NK cell lineage was not significantly affected (Fig. 1C). T-cell development in the thymus (as measured by CD4 and CD8 expression) was essentially normal in *Paxx*^−/−^ mice (Supplemental Fig. S3B), although they did exhibit a small increase in the number and percentage of mature B cells within the bone marrow (Supplemental Fig. S3C; data not shown). To test whether *Paxx*^−/−^ mice were capable of mounting a proper immune response, we immunized *Paxx*^−/−^ and wild-type littermates with purified fragment C of tetanus toxin and found that immune responses as measured by total immunoglobulin (Ig), IgG1, and IgG2a antibody titers were similar in both (Supplemental Fig. S3D). Furthermore, class switch recombination (CSR) was normal in the absence of PAXX (Supplemental Fig. S3E). As PAXX is important for efficient DSB repair in human cells (Craxton et al. 2015; Ochi et al. 2015; Xing et al. 2015), we treated *Paxx*^−/−^ mice with IR. Following whole-body IR treatment, we observed significantly increased lethality in *Paxx*^−/−^ mice when compared with wild-type controls, although this was not as dramatic as that observed in *Atm*^−/−^ mice (Fig. 1D; Barlow et al. 1996). Taken together, these data implied that although PAXX is important for effective DSB repair, it does not play an essential role in mammalian development and is unlikely to function as a strong tumor suppressor. In addition, *Paxx*^−/−^ mice showed only a mild reduction in lymphocyte numbers and displayed no overt immunodeficiency phenotype. In this regard, *Paxx*^−/−^ mice most closely resemble *Xlf*^−/−^ mice (Li et al. 2008) rather than mice deficient in other core NHEJ factors.

### Paxx loss is epistatic with Ku, Lig4, and Atm deficiency

Genetic crosses between NHEJ mutant mice and with other DNA repair-deficient mouse models have revealed both synthetic viability (e.g., *Ku80*^−/−^ *Lig4*^−/−^ mice) and synthetic lethality (e.g., *Atm*^−/−^ *Xlf*^−/−^ mice) relationships (Karanjawala et al. 2002; Zha et al. 2011). Such genetic crosses can highlight antagonistic roles and/or functional redundancies between DNA repair factors and can help position such factors in NHEJ and other events. We therefore crossed our *Paxx*^−/−^ mice into *Ku80*-deficient, *Lig4*-deficient, *Atm*-deficient, or *Xlf*-deficient backgrounds.

**Figure 2.**
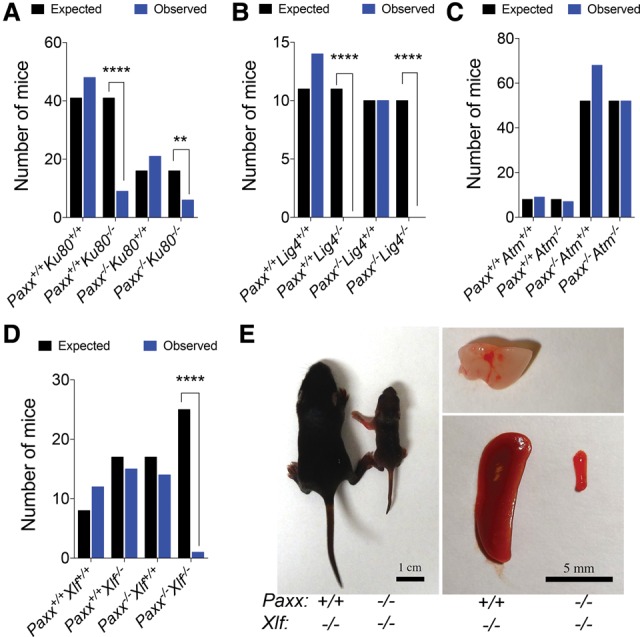
*Paxx/Xlf* deficiency leads to a synthetic-lethal phenotype. Mice were genotyped using ear snip biopsies, and expected versus observed numbers were used to calculate χ^2^. Bar graphs presenting selected genotype combinations are presented. (*A*) *Paxx/Ku80*-deficient mice are significantly underrepresented compared with wild-type mice (**P < 0.01) but are represented at ratios similar to that of *Ku80* deficiency alone ([****] *P* < 0.0001). (*B*) *Paxx* deficiency cannot save the *Lig4*-associated lethality. Neither *Lig4*^−/−^ nor *Paxx^−/−^ Lig4*^−/−^ mice are born. (****) *P* < 0.0001. (*C*) *Paxx* deficiency does not modify the phenotypes associated with *Atm* loss. Like *Atm*^−/−^ mice, *Paxx^−/−^ Atm*^−/−^ mice are born at the expected Mendelian frequencies. (*D*) Unlike *Paxx*^−/−^ or *Xlf*^−/−^ mice, *Paxx^−/−^Xlf*^−/−^ mice are not born at the expected frequencies. (****) *P* < 0.0001. (*E*) Photograph of a 10-d-old *Paxx^−/−^ Xlf*^−/−^ mouse as compared with a *Paxx^+/+^ Xlf*^−/−^ littermate control. Isolated spleens and thymus of the represented mice are shown. In the case of the *Paxx^−/−^ Xlf*^−/−^ mouse no thymus was identified.

*Paxx*^−/−^ *Ku80*^−/−^ mice were born at frequencies similar to *Ku80*^−/−^ mice and did not show any overt phenotypes compared with *Ku80*^−/−^ animals (Fig. 2A; Supplemental Table S2A), consistent with *Paxx* being epistatic with *Ku80* in regard to NHEJ. Notably, this was similar to what we observed with *Xlf*^−/−^ *Ku80*^−/−^ mice, which also closely resemble *Ku80*^−/−^ mice (Supplemental Fig. S4A,B). In contrast to *Ku80*^−/−^ mice, *Lig4*^−/−^ mice die during embryonic development in a manner associated with dramatically increased apoptosis in the CNS (Barnes et al. 1998; Frank et al. 1998; Gao et al. 1998). As *Ku80* loss rescues the lethality of *Lig4*^−/−^ mice (Karanjawala et al. 2002) and because PAXX functions in physical contact with Ku in NHEJ, we wondered whether PAXX loss would result in a similar rescue. However, multiple rounds of breeding did not produce any viable *Paxx*^−/−^ *Lig4*^−/−^ offspring, indicating that, unlike Ku80 loss, PAXX loss cannot rescue the embryonic lethality caused by LIG4 deficiency (Fig. 2B; Supplemental Table S2B). Next, we generated *Paxx*^−/−^ *Atm*^−/−^ mice to explore potential functional relationships between these two genes. Notably, in contrast to the relationship between *Atm* and *Xlf* (Zha et al. 2011), *Paxx*^−/−^ *Atm*^−/−^ mice displayed no additional phenotypes compared with *Atm*^−/−^ mice (Fig. 2C; Supplemental Fig. S5; Supplemental Table S2C), indicating that *Paxx* and *Atm* are epistatic for immune functions such as CSR and during development.

### Paxx loss is synthetic-lethal with Xlf deficiency

We also crossed *Paxx*^−/−^ mice with *Xlf*^−/−^ mice to examine potential functional relationships between these genes. Strikingly, these double-knockout mice were dramatically underrepresented, with only one *Paxx*^−/−^ *Xlf*^−/−^ mouse born out of 25 expected (Fig. 2D; Supplemental Table S2D). Furthermore, this mouse was born smaller and, at 5 d of age, was clearly distinguishable from its littermate controls (Supplemental Fig. S6A). By 10 d of age, this *Paxx*^−/−^ *Xlf*^−/−^ mouse failed to thrive and, at necropsy, exhibited a microspleen and a complete absence of a thymus, similar to what is observed in *Ku80*^−/−^ animals (Fig. 2E). In accord with these findings, there was a significant decrease in splenic cell counts relative to body weight, and, upon red blood cell lysis, no lymphocytes were recovered (Supplemental Fig. S6B,C). Taken together, these data indicated that while *Paxx* loss is epistatic with *Ku80*, *Lig4*, and *Atm* in terms of its developmental roles, it is synthetic-lethal in combination with *Xlf* loss.

**Figure 3.**
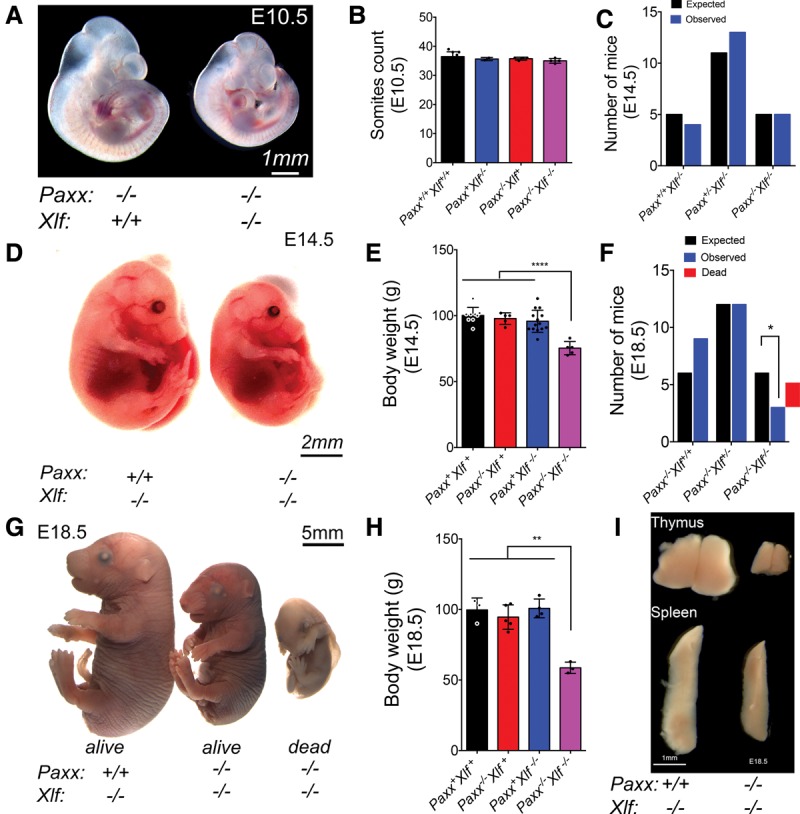
Embryonic lethality upon simultaneous loss of *Paxx* and *Xlf*. Embryos from timed matings were isolated at the indicated stage of embryonic development, imaged for morphological assessment, and genotyped by PCR from the yolk sacs or tail snips. (*A*) Representative images of *Paxx*^−/−^ and *Paxx^−/−^ Xlf*^−/−^ E10.5 littermate embryos. (*B*) Bar graph of somite counts from E10.5 dissected litters. (*C*) Bar graph (mean ± SD) of expected versus observed E14.5 embryos. (*D*) Representative images of *Paxx*^−/−^ and *Paxx^−/−^ Xlf*^−/−^ E14.5 littermate embryos. (*E*) Bar graph representing the percentage relative body weight as compared with control littermates at E14.5. (****) *P* < 0.0001, Student's *t* test. (*F*) Bar graph of expected versus observed E18.5 embryos. (*) *P* < 0.05, Student's *t* test. The red bar was added to represent the number of *Paxx^−/−^ Xlf*^−/−^ embryos found dead. (*G*) Representative images of *Xlf*^−/−^ (*left*) and *Paxx^−/−^ Xlf*^−/−^ E18.5 littermate embryos. (*H*) Bar graph (mean ± SD) representing the percentage relative body weight as compared with control littermates at E18.5. (**) *P* < 0.01, Student's *t* test. (*I*) Representative images of thymi and spleens of *Xlf*^−/−^ as compared with *Paxx^−/−^ Xlf*^−/−^ littermate E18.5 live embryos.

To determine when *Paxx*^−/−^ *Xlf*^−/−^ mice die during embryonic development, we established timed matings and performed embryo dissections starting at embryonic day 9.5 (E9.5) when *Paxx/Xlf* double mutants showed no gross abnormalities (Supplemental Fig. S7A). By E10.5, although double mutants were recovered at the expected frequencies, more than half of the *Paxx*^−/−^ *Xlf*^−/−^ embryos were smaller than their littermate controls (Fig. 3A; Supplemental Fig. S7B). Nevertheless, the *Paxx/Xlf* double mutants were not developmentally delayed at this stage, showing somite numbers similar to those of wild-type, *Paxx*^−/−^, or *Xlf*^−/−^ embryos (Fig. 3B), thus hinting at a possible cell fate abnormality. Similarly, at E14.5, double-mutant embryos were obtained at the expected Mendelian frequencies (Fig. 3C; Supplemental Table S3A) but were smaller than controls (Fig. 3D,E). By E18.5, *Paxx*^−/−^ *Xlf*^−/−^ double mutants were no longer obtained at the expected frequencies, and a significant proportion of them was found to have died (Fig. 3F; Supplemental Table S3B). A few were viable but showed reduced body weight, much smaller spleens, and a drastic involution of the thymus (Fig. 3G–I), thus highlighting a requirement for either PAXX or XLF in lymphatic organ development and for life for more than a few days postnatally.

**Figure 4.**
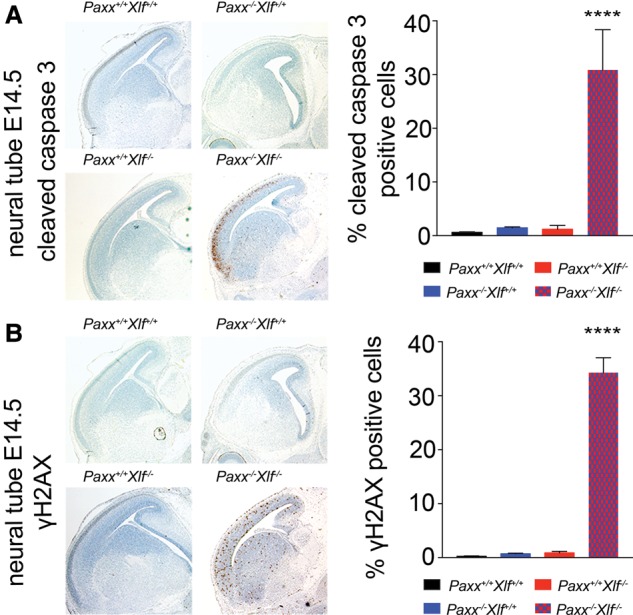
Increased genomic instability and apoptosis in the CNS of *Paxx^−/−^ Xlf*^−/−^ double-mutant embryos. Representative images (5× magnification) depicting immunohistochemical staining for cleaved caspase 3-positive apoptotic cells (*A*) and γH2AX-positive cells (*B*) in the neural tubes of E14.5 embryos. Bar graphs (mean ± SD) present the percentage of cleaved caspase 3-positive (*A*) and pan-nuclear γH2AX-positive (*B*) cells of the representative genotypes. More than 500 cells per embryo were counted. *n* ≥ 3 per genotype. Statistical analysis was performed using one-way ANOVA. (****) *P* < 0.0001, Dunnett's multiple comparisons test.

To determine the cause of embryonic death in *Paxx*^−/−^ *Xlf*^−/−^ mice, we looked for markers of genomic instability (phosphorylated histone H2AX; γH2AX) and apoptosis (cleaved caspase 3) in E10.5 and E14.5 embryos via immunohistochemistry. At both E10.5 and E14.5, we consistently observed an increase in γH2AX-positive cells in the CNS of the *Paxx*^−/−^ *Xlf*^−/−^ double-mutant mice as compared with littermate controls (Fig. 4A,B; Supplemental Fig. S7C). This was associated with increased apoptosis, as evidenced by the accumulation of cleaved caspase 3, especially in the cortical plate region of the cerebral cortex (Fig. 4B; Supplemental Fig. S7C,D). In contrast, significant increases in these markers were not detected in most other tissues (Supplemental Fig. S8A,B). Together, these findings highlighted a critical requirement for *Paxx* or *Xlf* for development of not only lymphatic organs but also the CNS. We thus conclude that lack of effective maintenance of genome stability during development results in increased cell death and embryonic lethality in the absence of both PAXX and XLF.

In this study, we described the generation of PAXX-deficient mice. We found that while *Paxx*^−/−^ mice are radiosensitive, they are born at Mendelian ratios and display no overt immunodeficiency or developmental defects. In this regard, *Paxx*^−/−^ mice are reminiscent of *Xlf*^−/−^ mice, although the immunological phenotype of the latter is slightly more pronounced (Li et al. 2008). Furthermore, our data support a model in which PAXX functions in NHEJ in a manner connected to and dependent on Ku because *Ku80^−/−^ Paxx*^−/−^ double-mutant mice were essentially identical to *Ku80*^−/−^ mice. Furthermore, we found that PAXX loss could not rescue the embryonic lethality phenotype of *Lig4*^−/−^ mice. This was expected based on previous cell-based studies and biochemical analyses of PAXX, which showed that PAXX functions downstream from Ku in the NHEJ process, being recruited to Ku-bound DNA ends by direct binding of the PAXX C terminus to Ku (Ochi et al. 2015; Xing et al. 2015).

Perhaps surprisingly, we found that combined PAXX and XLF deficiency is lethal in mice. This was unexpected based on our previous results in human cells, where we found that depletion of XLF in *PAXX*^−/−^ RPE-1 cells caused no additional radiosensitivity (Ochi et al. 2015). Interestingly, we were able to recapitulate this epistasis in mouse embryonic fibroblasts (MEFs) derived from our *Paxx*/*Xlf* single-knockout and double-knockout mice exposed to IR and the radiomimetic drug phleomycin (Supplemental Fig. S9A–C). Due to the extensive cell death in lymphoid progenitors in developing embryos, we were unable to isolate B cells from *Paxx^−/−^ Xlf*^−/−^ mice to examine their radiosensitivity in vitro. However, recent data using *Paxx*/*Xlf* single-knockout and double-knockout v-Abl transformed pro-B cells derived from wild-type mice demonstrated that, in this cell lineage as in the developing CNS, a synthetic relationship between PAXX and XLF exists in terms of cellular sensitivity to IR as well as in the process of V(D)J recombination (Kumar et al. 2016; Lescale et al. 2016b). Thus, there appears to be a tissue-specific requirement for PAXX and XLF: In some cells, both PAXX and XLF are required for NHEJ, but, in others, either one or the other suffices to a major degree. In this regard, we note that tissue-specific requirements for XLF in mice have been demonstrated previously (Li et al. 2008).

Since the generation of XLF-deficient mice was first reported (Li et al. 2008), it has been unclear why the phenotype of these mice is relatively mild compared with mice in which other core NHEJ factors are mutated given that XLF is important for DSB repair by NHEJ in cells (Ahnesorg et al. 2006; Buck et al. 2006). To help explain these findings, functional redundancy with other factors was suggested and, in due course, demonstrated by XLF loss being shown to display nonepistatic or synthetic-lethal relationships with loss of components of the ATM–H2AX–53BP1 pathway or with C-terminal mutants of the V(D)J recombinase protein RAG2 (Zha et al. 2011; Liu et al. 2012; Oksenych et al. 2012; Lescale et al. 2016a). Our data reveal that PAXX and XLF also share one or more redundant functions, as, unlike single mutants, mice lacking both proteins display severe growth defects, extensive cell death in the CNS, and an almost complete block to lymphocyte development similar to that of *Xrcc4*^−/−^ or *Lig4*^−/−^ mice. Interestingly, unlike the situation when combining XLF and ATM loss, combined loss of PAXX and ATM did not lead to a phenotype more severe than that of mice lacking ATM alone. Considering our findings, it will be of interest to further explore the overlapping and nonoverlapping functions of PAXX and XLF in NHEJ during development, define the basis for the apparent tissue-specific effects of their combined loss, and establish how PAXX might operate in the context of ATM-mediated DSB repair processes.

## Materials and methods

### Animals

Care and use of all mice used to generate data for this protocol were carried out in accordance with UK Home Office regulations, UK Animals (Scientific Procedures) Act of 2013. *Paxx*-deleted (*C57BL/6NTac-Paxx*^em1Gb^) mice were generated using CRISPR/Cas9. *Ku80* (*B6.129-Xrcc5^tm1Nus^/J*; stock no: 004361), *Lig4 (B6;129S6-Lig4*^*tm1Fwa/Kvm*^; stock no: 006482*)*, and *Atm* (*129S6-Atm^tm1Awb^/J*); stock no. 008671) knockout mice were imported from Jackson Laboratories. *Xlf* (*129S6/SvEvTac-Nhej1*^*tm1Fwa*^) knockout mice (Li et al. 2008) were imported from the laboratory of Dr. Shan Zha at Columbia University. The double-mutant combinations were maintained on the selected mixed backgrounds. For analysis of embryonic development, timed matings were performed. Noon of the day of vaginal plug detection was defined as E0.5.

### IR treatment

For analysis of survival upon IR, mice were subjected to 5 Gy of γ irradiation using a sealed ^137^Cs source γ irradiator (CIS-IBL 437C). Subsequently, mice were monitored by daily weighing for up to 30 d or until reaching humane endpoint criteria based on the clinical disease scoring system detailed in the Supplemental Material.

### Histology, immunohistochemistry, and immunoblotting

All major organs were isolated following euthanasia and then fixed in 10% formalin overnight. Embryos were dissected on cold PBS and fixed in 4% paraformaldehyde. On the second day, the fixed organs were transferred to 70% ethanol, placed in cassettes, and embedded in paraffin, and serial 5-µm sections were collected on Superfrost Plus slides (Thermo Fisher Scientific) using a Leica microdissection system (LMD7000). For immunohistochemistry apoptosis and DNA damage, quantitation was done using antibodies against cleaved caspase 3 (Abcam, ab13847) or γH2AX (clone JBW301; Millipore), respectively. For Western blotting, antibodies against PAXX (Abcam, ab126353) and β-actin (Sigma, A5316) were used. Histology images were obtained using an Aperio Scanscope (Aperio Technologies). Statistical analysis was performed using Prism 7 (Graphpad). Student's *t* tests were always two-tailed.

## Supplementary Material

Supplemental Material
