# Supplementary material for: Synthetic lethality between PAXX and XLF in mammalian development
Source: Genes Dev. 2016 Oct 1;30(19):2152–7. doi: 10.1101/gad.290510.116 (PMC5088564; doi:10.1101/gad.290510.116)
Supplement: Supplemental Material [file supp_30_19_2152__index.html]

Supplemental Material 

# Synthetic lethality between PAXX and XLF in mammalian development

## Supplemental Material

- Supplemental\_Fig\_S1.pdf
- Supplemental\_Fig\_S8.pdf
- Supplemental\_Table\_S2.pdf
- Supplemental\_Fig\_S6.pdf
- Supplemental\_Material.pdf
- Supplemental\_Fig\_S4.pdf
- Supplemental\_Fig\_S2.pdf
- Supplemental\_Fig\_S9.pdf
- Supplemental\_Table\_S3.pdf
- Supplemental\_Fig\_S7.pdf
- Supplemental\_Table\_S1.pdf
- Supplemental\_Fig\_S5.pdf
- Supplemental\_Fig\_S3.pdf
