# Supplementary material for: Synthetic lethality between PAXX and XLF in mammalian development
Source: Genes Dev. 2016 Oct 1;30(19):2152–7. doi: 10.1101/gad.290510.116 (PMC5088564; doi:10.1101/gad.290510.116)

Figure S1. Tyrosinase knockout strategy used to select for *Paxx*$^{-/-}$ chimeric mice. A) In order to allow for color-based selection of the *Paxx* deletion, a gRNA targeting exon 1 (*tyr1*) within the tyrosinase locus was chosen. Sequence representation of the targeted locus with the gRNA (Tyr1) represented. B) Homozygous disruption of the tyrosinase locus results in coat color change of C57BL/6NTac mice from black to white as a control for successful injection and function of CRISPR/Cas9 reagents. Represented white color pups are *Tyr*$^{-/-}$ *Paxx*$^{-/-}$ mutant chimeras; dark color pups have no modification of the *Paxx* locus.

Balmus_Supplemental_Fig_S1

A

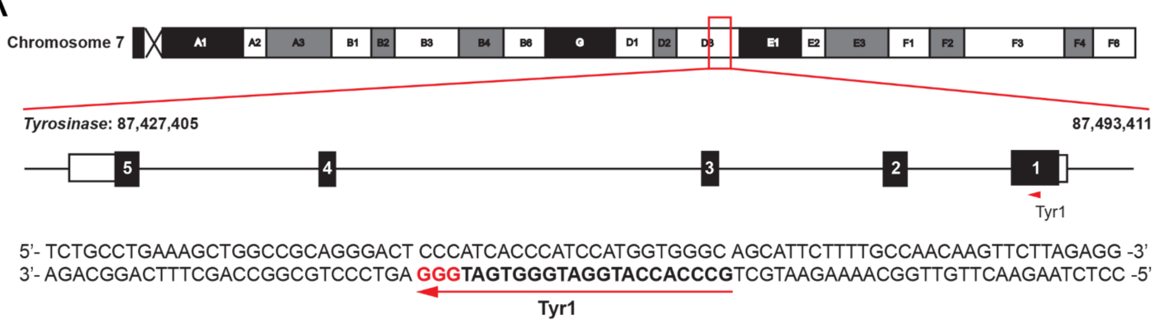

B

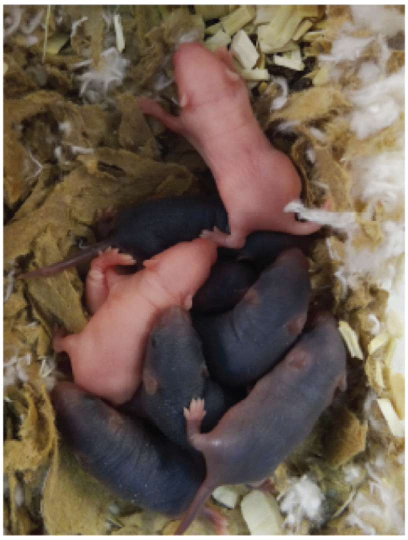

Supplement: Supplemental Material [file supp_30.19.2152_Supplemental_Fig_S1.pdf]
