# Supplementary material for: Synthetic lethality between PAXX and XLF in mammalian development
Source: Genes Dev. 2016 Oct 1;30(19):2152–7. doi: 10.1101/gad.290510.116 (PMC5088564; doi:10.1101/gad.290510.116)

Figure S8. *Paxx*$^{-/-}$ *Xlf*$^{-/-}$ embryos show mild genomic instability and no increased apoptosis in other organ systems. A) Representative images of γH2AX-positive cells in the lung, heart, liver and skin of E14.5 embryos of the representative genotypes; size bar 10 µm. Bar graphs (mean ± SD) presenting the % pan-nuclear γH2AX-positive cells. Three random 40X fields of view were counted (n≥3). One-way ANOVA (Dunnett's multiple comparisons test; *p<0.01; **p<0.001) statistical analysis was performed. B) Representative images of cleaved caspase 3-positive cells in the lung, heart, liver and skin of E14.5 embryos of the representative genotypes; size bar 10 µm. Bar graphs (mean ± SD) presenting the % cleaved caspase 3-positive cells. Three random 40X fields of view were counted (n≥3). One-way ANOVA (Dunnett's multiple comparisons test) statistical analysis was performed.

Balmus_Supplemental_Fig_S8

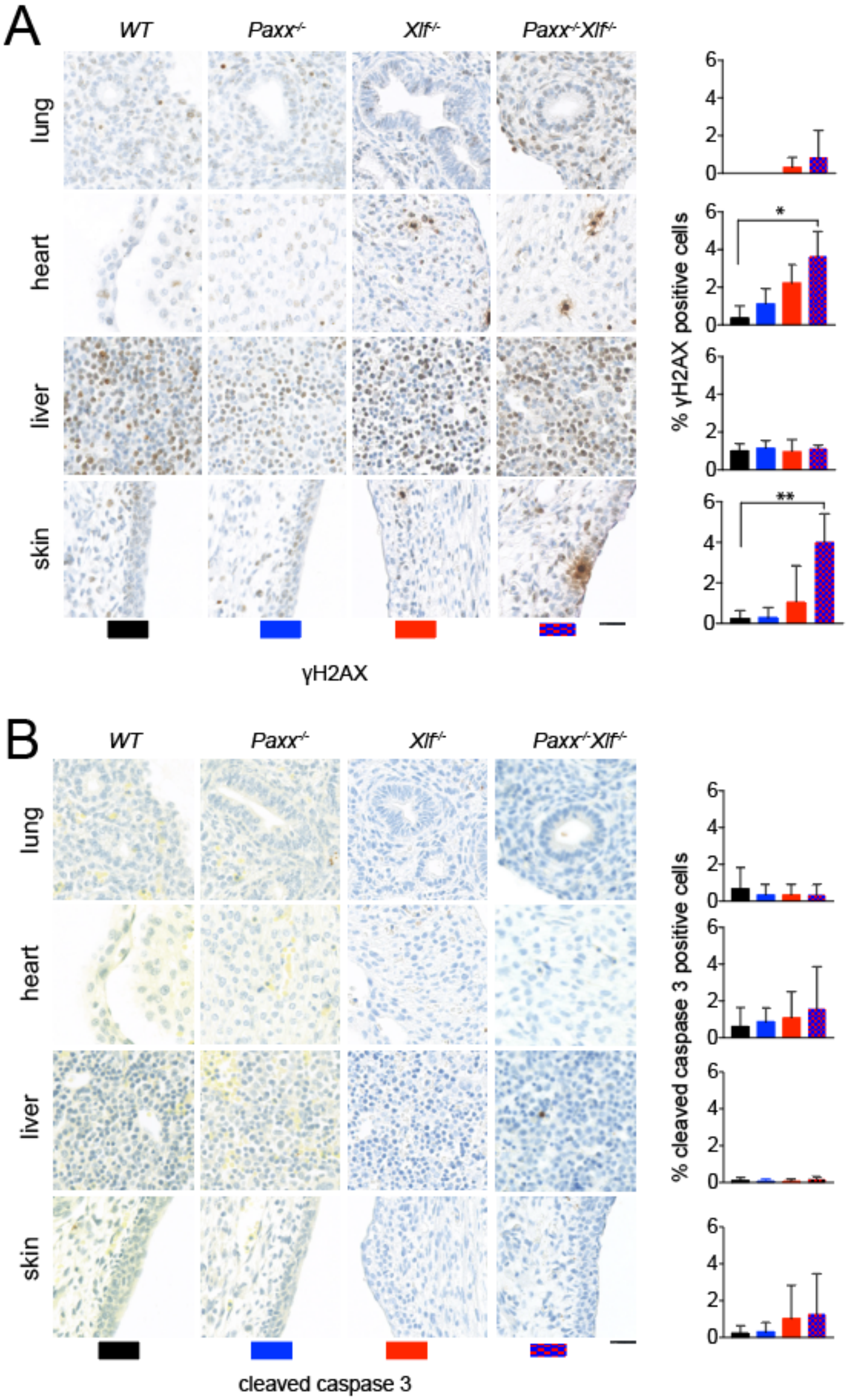

Supplement: Supplemental Material [file supp_30.19.2152_Supplemental_Fig_S8.pdf]
