# Supplementary material for: Synthetic lethality between PAXX and XLF in mammalian development
Source: Genes Dev. 2016 Oct 1;30(19):2152–7. doi: 10.1101/gad.290510.116 (PMC5088564; doi:10.1101/gad.290510.116)
Supplement: Supplemental Material [file supp_30.19.2152_Supplemental_Table_S2.pdf]

Supplemental Table S2. *Paxx* epistasis analysis.

Mice were genotyped by PCR analysis of tail DNA at 2 weeks of age. Expected values represent the combined Mendelian percentages and numbers from several crosses involving different parental genotypes of the *Paxx/Ku80* cross (A), *Paxx/Lig4* cross (B) *Paxx/Atm* cross (C) and *Paxx/Xlf* cross (D). The significant values of observed and expected genotype frequencies were calculated using the $\chi 2$ and the representative data was graphed in Figure 2.

Balmus_Supplemental_Table_S2

A

Genotype		% Expected	% Observed	# Expected	# Observed
Paxx$^{+/+}$	*Ku80*$^{+/+}$	15	18	41	48
	Ku80$^{+/-}$	30	38	82	103
	Ku80$^{-/-}$	15	3	41	9
Paxx$^{+/-}$	*Ku80*$^{+/+}$	4	4	10	12
	Ku80$^{+/-}$	8	10	21	27
	Ku80$^{-/-}$	4	1	10	3
Paxx$^{-/-}$	*Ku80*$^{+/+}$	6	8	16	21
	Ku80$^{+/-}$	12	15	32	40
	Ku80$^{-/-}$	6	2	16	6
**TOTAL**		**100**	**100**	**269**	**269**

C

Genotype		% Expected	% Observed	# Expected	# Observed
Paxx$^{+/+}$	*Atm*$^{+/+}$	3	3	9	8
	Atm$^{+/-}$	6	9	17	28
	Atm$^{-/-}$	3	2	9	7
Paxx$^{+/-}$	*Atm*$^{+/+}$	6	5	17	16
	Atm$^{+/-}$	11	9	35	26
	Atm$^{-/-}$	6	4	17	12
Paxx$^{-/-}$	*Atm*$^{+/+}$	17	23	52	68
	Atm$^{+/-}$	33	38	104	111
	Atm$^{-/-}$	17	12	52	36
**TOTAL**		**100**	**100**	**312**	**312**

B

Genotype		% Expected	% Observed	# Expected	# Observed
Paxx$^{+/+}$	*Lig4*$^{+/+}$	10	5	11	14
	Lig4$^{+/-}$	20	12	22	33
	Lig4$^{-/-}$	10	0	11	0
Paxx$^{+/-}$	*Lig4*$^{+/+}$	6	3	7	8
	Lig4$^{+/-}$	13	6	14	15
	Lig4$^{-/-}$	6	0	7	0
Paxx$^{-/-}$	*Lig4*$^{+/+}$	9	4	10	10
	Lig4$^{+/-}$	18	12	20	31
	Lig4$^{-/-}$	9	0	10	0
**TOTAL**		**100**	**100**	**111**	**111**

D

Genotype		% Expected	% Observed	# Expected	# Observed
Paxx$^{+/+}$	*Xlf*$^{+/+}$	5	6	8	12
	Xlf$^{+/-}$	9	9	17	16
	Xlf$^{-/-}$	9	8	17	15
Paxx$^{+/-}$	*Xlf*$^{+/+}$	9	13	17	25
	Xlf$^{+/-}$	18	18	34	34
	Xlf$^{-/-}$	18	20	34	37
Paxx$^{-/-}$	*Xlf*$^{+/+}$	9	8	17	14
	Xlf$^{+/-}$	9	17	17	32
	Xlf$^{-/-}$	14	1	25	1
**TOTAL**		**100**	**100**	**186**	**186**
