# Supplementary material for: Synthetic lethality between PAXX and XLF in mammalian development
Source: Genes Dev. 2016 Oct 1;30(19):2152–7. doi: 10.1101/gad.290510.116 (PMC5088564; doi:10.1101/gad.290510.116)

Figure S6. ***Paxx*** $^{-/-}$ ***Xlf*** $^{-/-}$ **mice show a synthetic lethal phenotype.** A) Photograph of 5 days old Paxx/Xlf mutants of the selected genotypes. Arrow indicates *Paxx* $^{-/-}$ *Xlf* $^{-/-}$ double mutant mouse. *Paxx* $^{+}$ genotype refers to a combination of *Paxx* $^{+/+}$ and *Paxx* $^{+/-}$ mice. B) Bar graphs presenting the body weight of the *Paxx* $^{-/-}$ *Xlf* $^{-/-}$ mouse as compared to age matched *Paxx* $^{+/+}$ *Xlf* $^{+/+}$ (n=4) and *Paxx* $^{+/+}$ *Xlf* $^{-/-}$ (n=4) controls at 10 days of age, as well as the weight of the spleen relative to their body weight. C) Bar graphs presenting the spleen cell counts of the *Paxx* $^{-/-}$ *Xlf* $^{-/-}$ mouse as compared to age matched *Paxx* $^{+/+}$ *Xlf* $^{+/+}$ (n=4) and *Paxx* $^{+/+}$ *Xlf* $^{-/-}$ (n=4) controls at 10 days of age, before or after red blood cell (RBC) lysis (log10 scale).

Balmus_Supplemental_Fig_S6

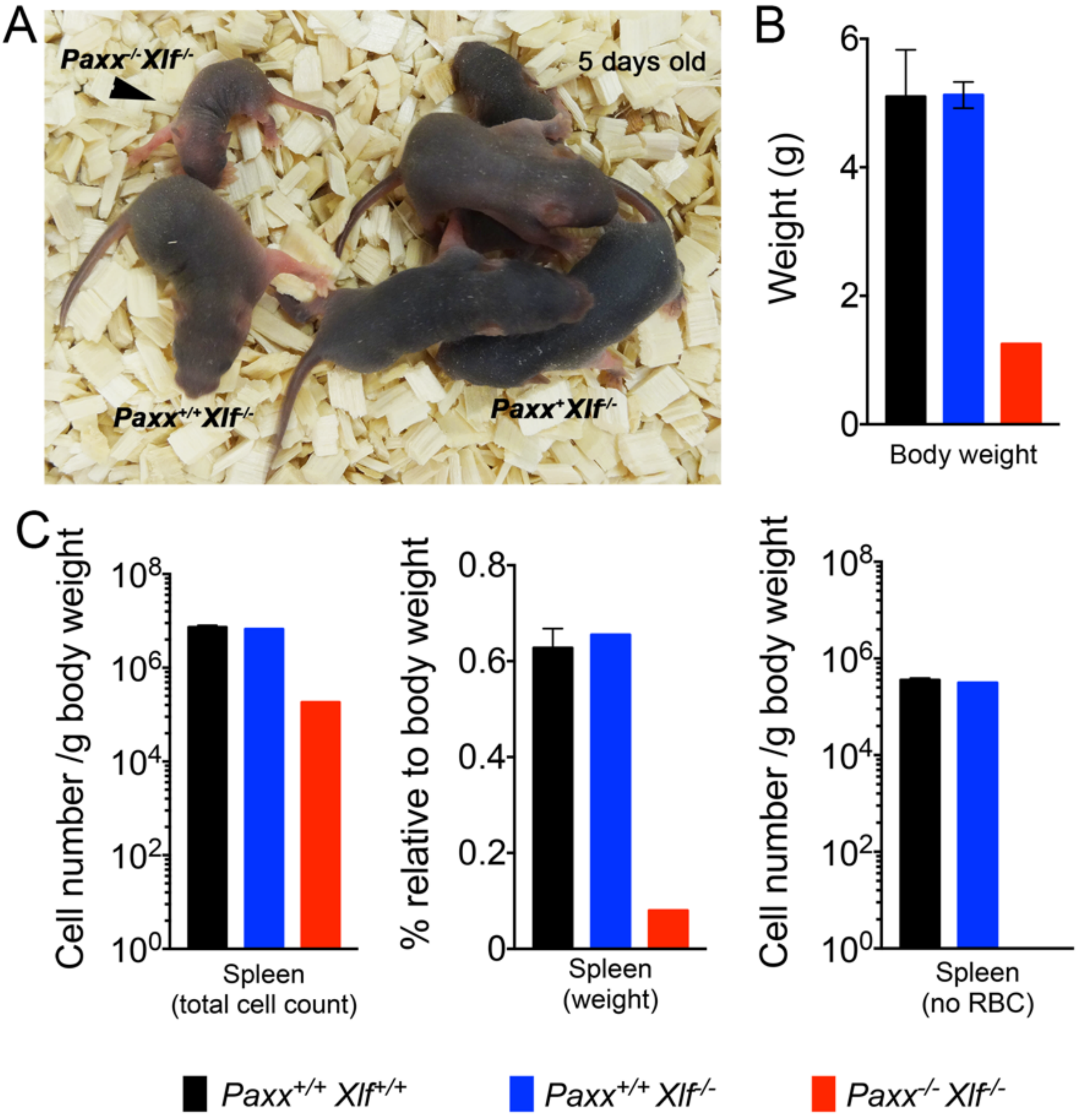

Supplement: Supplemental Material [file supp_30.19.2152_Supplemental_Fig_S6.pdf]
