# Supplementary material for: Synthetic lethality between PAXX and XLF in mammalian development
Source: Genes Dev. 2016 Oct 1;30(19):2152–7. doi: 10.1101/gad.290510.116 (PMC5088564; doi:10.1101/gad.290510.116)
Supplement: Supplemental Material [file supp_30.19.2152_Supplemental_Material.pdf]

Supplemental material

Supplemental materials and methods

Animal housing

Mice were maintained in a specific pathogen-free unit on a 12-hour light:12-hour dark cycle with lights off at 19:30 and no twilight period. The ambient temperature was 21 ± 2 °C, and the humidity was 55 ± 10%. Mice were housed using a stocking density of 3–5 mice per cage (overall dimensions of caging: 365 × 207 × 140 mm (length × width × height), floor area 530 cm^2) in individually ventilated caging (Tecniplast, Sealsafe 1284L) receiving 60 air changes per hour. In addition to Aspen bedding substrate, standard environmental enrichment of two Nestlets, a cardboard fun tunnel and three wooden chew blocks are provided. Mice were given water and diet ad libitum.

CRISPR/Cas9 target sites and vector construction

CRISPR target sites were identified using http://crispr.mit.edu/. The strategy to generate mouse lines carrying the *Paxx* deletion is shown in Fig. 1A and Fig. S1. Briefly, one pair of gRNAs was selected in the 5' UTR of *Paxx* and a second pair of gRNAs in the 3' UTR. A DNA template, complementary to part of the 5' and 3' regions (LHA and RHA, respectively) directly adjacent to the gRNA sites was included to aid in end-to-end fusion. Indicated primers were used to determine gene status. A gRNA against the *Tyr* locus (*tyr1*) was co-injected to allow for color-based selection of *Paxx* deletions. The selected gRNA sequences were cloned into pU6 gRNA cloning vector (a gift from Dr. W. Skarnes) using *Bsa*I. For Cas9 mRNA production, pX330-U6-Chimeric_BB-CBh-hSpCas9 was modified to contain the T7 promoter. pX330-U6-Chimeric_BB-CBh-hSpCas9 (Addgene plasmid # 42230) was a gift from Dr. F. Zhang (Cong et al. 2013). The integrity of all plasmids was confirmed by

DNA sequencing. The template oligodeoxynucleotides were synthesized as desalted ssDNA oligonucleotides from Integrated DNA Technologies (IDT). The collapse oligonucleotide sequence was GCT AAA CCC CGC CCT TTA GTC AGC CTC TTA CTT AAT GCC TGG TAC CCG GAA ACA ATG TCA ACC TTG AGT ACC GCC CAT CGC CTC GTC CTT CCG TTC AAA TAA ACT GCT CCC TCG GGG AGA GAT GCT AGG CCA GAC TTG AGT TGT GAA GAT TGG CCT TGG GGA GGG GGC ACG AGA AGA CCC CTC TG.

gRNA production and PCR genotyping

Primers used for producing the individual gRNAs were as follows:

T7-FSgRNA1 5'UTR *Paxx*

GTCTC**TAATACGACTCACTATAGGG**CCGAGTTTGTACTTCTGCGCGTTTTAGAGCTAGA

T7-RSgRNA1 5'UTR *Paxx*

GTCTC**TAATACGACTCACTATAGGG**CTAAGGTGTTCGCTCGGCGGGTTTTAGAGCTAGA

T7-FSgRNA2 3'UTR *Paxx*

GTCTC**TAATACGACTCACTATAGGG**GTGGTCCTGCTGTTGGGCAAGTTTTAGAGCTAGA

T7-RSgRNA2 3'UTR *Paxx*

GTCTC**TAATACGACTCACTATAGGG**CAAGGGGTGTCTCTGATTAGGTTTTAGAGCTAGA

The T7 sequence is highlighted in bold, the underlined sequence represents the gRNA specific sequence targeting either the 5' UTR or 3' UTR of mouse *Paxx*, followed by the start of the tracrRNA. PCR product size was verified by agarose gel electrophoresis as a unique 120-bp product (guide + tracrRNA), and PCR purified. Next, 100 nM of each product was used as a template for *in vitro* transcription using the T7 MEGAshortscript kit (Ambion AM1354) according to the manufacturer's instructions. Finally, the products were purified using the MEGAclear RNA purification kit (Ambion AM1908) and eluted in RNase-free water. The quality of the RNA was analyzed using Agilent RNA 6000 Nano kit (Agilent Technologies, 2100 Bioanalyzer) and Qubit RNA HS assay kit (Life Technologies). The PCR

genotyping oligonucleotides as represented in Fig. 1A were: *Paxx* EX4 **R1**- 5’ CAT ACA GTA CCT GCC AGC CG 3’; *Paxx* EX4 **F2** - 5’ GTT CCA GTT AGG GAG GCC AT 3’; *Paxx* **F1** - 5’TCA ACC TTG AGT ACC GCC 3’; *Paxx* **R2** - 5’ GCT GCC TGC CTT AAG ACC TA 3’.

Cytoplasmic injections

For cytoplasmic injections, an injector using a positive balance pressure such as an Eppendorf Femtojet was connected to a microinjection tip filled with the CRISPR material. The microinjection tip was steadily advanced towards the opposite side of the zygote, which was anchored by the holding pipette until the micropipette passed through the plasma membrane. The pipette was then drawn back into the cytoplasm, the CRISPR mix delivered and the micropipette immediately withdrawn. A successful injection was indicated by visible movement in the cytoplasm.

Immune assays

Retro-orbital blood was collected into EDTA-coated tubes and complete blood counts were determined using a scil Vet ABC system. Single cell suspensions from spleen and thymus were prepared using frosted ends of microscope slides in FACS buffer (PBS, 0.5-1% bovine serum albumin [BSA]). Bone marrow was flushed from the femur with FACS buffer. Red blood cells were lysed from spleen and bone marrow samples by the addition of 1 ml 1x Pharm Lyse (BD Biosciences) for 90 seconds at room temperature then stopped by the addition of 10 ml FACS buffer. Cell numbers were determined using a Moxi cell counter. Samples were blocked with 1 μg Mouse FC block (2.4G2, BD Biosciences) for 10 minutes at 4 °C and then titrated antibody cocktails to give saturating binding were added for 30 minutes at 4 °C. Samples were washed and viability was determined by staining for 10 minutes at

room temperature with LIVE/DEAD Fixable Blue (Thermo Fisher Scientific) prior to acquisition on a BD LSRII instrument using 96-well plates via a high throughput sampler. For bone marrow B cell development B cells were identified as B220+ and subsequently separated into pre-pro B cells (CD24low/neg and BP-1neg) and developing B cells (CD24hi). Developing B cells were classified into five subsets on the basis of surface IgM and IgD expression. For the CSR assay, splenic B cells from 6-8 week-old mice were isolated using CD19 microbeads (Miltenyi) and cultured for 96 hours in RPMI medium (Thermo Fisher Scientific) supplemented with α-CD40 antibody (1 g/ml, Miltenyi) and IL-4 (10 ng/ml, Miltenyi) or LPS (50 μg/ml, Sigma-Aldrich) and IL-4 to switch to IgG1, and LPS to switch to IgG2b or IgG3. Cells were assayed for switching by flow cytometry on a Canto II (BD Biosciences) and data were analyzed using FlowJo (TreeStar). For the analysis of humoral immune responses mice were immunized subcutaneously with 100 μg of purified fragment C of tetanus toxin on days 0, 7 and 21. On day 28 post-immunization, 200 μl of blood was collected from the tail vein. Serum was collected and stored at -20 ^{o}C prior to analysis. For analysis, microtiter plates (Maxisorp, Nunc, Denmark) were coated overnight at 4 ^{o}C with 50 μl carbonate buffer (pH 9.6) containing recombinant fragment C (2 μg/mL). The buffer was subsequently removed by plate inversion and the plates were washed once with PBS containing 0.01% Tween-20 (wash buffer). Plates were blocked with 100 μl 3% BSA in PBS (blocking solution) at room temperature for 1 hour. Plates were then washed once with wash buffer before sera from the individual mice were added as follows: 3 μl of serum was added to 27 μl of PBS + 1% BSA (antibody buffer). Then, 12.5 μl of the serum dilution was added, together with 112.5 μl antibody buffer, to an ELISA plate (1:500 mouse serum dilution). The plates, which also contained control wells with naive serum or PBS alone, were incubated for 1 hour at 37 ^{o}C. The plates were subsequently washed with wash buffer, after which 100 μl (1:1000 in antibody buffer) horse radish peroxidise (HRP) conjugated Ig, IgG1, IgG2a

specific antibody was added and incubated for 1 hour at 37 ºC. The antibodies were removed and the plates washed with washing buffer. Finally, 50 µl of o-phenylenediamine substrate (Sigma-Aldrich) was added to the plate wells and incubated for 15 minutes at room temperature. The reaction was stopped by addition of 25 µl 3 M sulphuric acid. Absorbances were measured at 490 nm and titers were determined arbitrarily as the reciprocal serum dilution corresponding to an optical density of 0.3.

Cell culture and survival assays

MEFs were prepared from E13.5 embryos, following timed matings between *Paxx*$^{+/-}$ *Xlf*$^{+/-}$ mice as previously described (Balmus et al. 2012). Briefly, embryos were dissected from the deciduum, mechanically disrupted and cultured in DMEM supplemented with 10% fetal bovine serum, 1 mM L-glutamine, 0.1 mM minimal essential medium nonessential amino acids, 100 µg/ml streptomycin sulfate and 100 U/ml penicillin. Initial plating was defined as passage zero (p0), and cells were subsequently maintained on a 3T3 protocol. At passage 3 cells were immortalized using an SV40 lentiviral construct as previously described (Xu 2005). Cellular survival was assessed by plating MEFs in six-well dishes at 1×10^5 cells per well in triplicate. Twenty-four hours later, cells were washed once with PBS after which normal growth medium was added. The cells were then exposed to various doses of IR or treated with the radiomimetic chemical, phleomycin. At the indicated time points cells were stained with trypan blue (1:1) and counted using an automated counter.

Ionizing radiation (IR) end point scoring system

All experiments were performed in accordance with relevant local animal research regulations and ARRIVE guidelines (Kilkenny et al. 2010). For the IR survival experiments we used a point-based scoring system previously reported (Nunamaker et al. 2013). Because

there is a reported age influence on IR sensitivity (Storer 1966), we irradiated only above 4 months of age (>12 weeks). Mice of both sexes were used as no sex bias has been previously reported. C57BL/6NTac mice were provided with antibiotics (neomycin, Sigma N6386, 0.4 mg/ml) and weighed, 24 hours prior to irradiation. Upon whole body irradiation with a sub-lethal dose of IR (5-6 Gy), mice were routinely weighed 3 times a week. If mice reached an additive score of ≥3 (Nunamaker et al. 2013), mice were weighed daily. Mice were euthanized when they reached a score ≥7 or >20% body weight loss.

Neutral comet assay

Neutral comet assays were performed as previously described (Ochi et al. 2015).

References

Balmus G, Zhu M, Mukherjee S, Lyndaker AM, Hume KR, Lee J, Riccio ML, Reeves AP, Sutter NB, Noden DM, et al. 2012. Disease severity in a mouse model of ataxia telangiectasia is modulated by the DNA damage checkpoint gene Hus1. *Human Molecular Genetics* **21**: 3408–3420.

Cong L, Ran FA, Cox D, Lin S, Barretto R, Habib N, Hsu PD, Wu X, Jiang W, Marraffini LA, et al. 2013. Multiplex genome engineering using CRISPR/Cas systems. *Science* **339**: 819–823.

Kilkenny C, Browne WJ, Cuthill IC, Emerson M, Altman DG. 2010. Improving bioscience research reporting: the ARRIVE guidelines for reporting animal research. *PLoS Biol* **8**: e1000412.

Nunamaker EA, Anderson RJ, Artwohl JE, Lyubimov AV, Fortman JD. 2013. Predictive observation-based endpoint criteria for mice receiving total body irradiation. *Comp Med* **63**: 313–322.

Storer JB, Chapter 22, Biology of the Laboratory Mouse, Earl L. Green, *Editor, Dover Publications, INC., NEW YORK 1966*

Xu J. 2005. Preparation, culture, and immortalization of mouse embryonic fibroblasts. *Curr Protoc Mol Biol* Chapter 28: Unit 28.1–28.1.8.
