# Supplementary material for: Synthetic lethality between PAXX and XLF in mammalian development
Source: Genes Dev. 2016 Oct 1;30(19):2152–7. doi: 10.1101/gad.290510.116 (PMC5088564; doi:10.1101/gad.290510.116)
Supplement: Supplemental Material [file supp_30.19.2152_Supplemental_Fig_S4.pdf]

Figure S4. ***Xlf$^{-/-}$ Ku80$^{-/-}$*** **mice are born at expected frequencies and show a** ***Ku80$^{-/-}$*** **like phenotype.** A) Mice were genotyped using ear-snip biopsies and expected *vs.* observed numbers used to calculate chi-square. Bar graphs presenting selected genotype combinations are presented. B) Images of Xlf/Ku80 mutant male mice of representative genotypes at 8 weeks of age; representative photographs of spleens and the thymus.

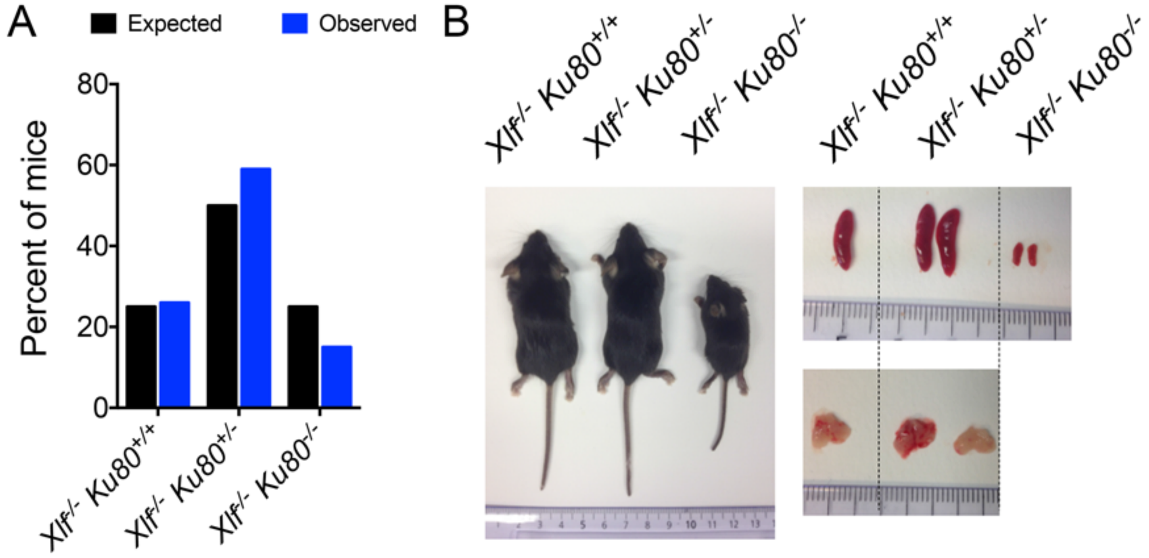
A
Expected
Observed
Percent of mice
80
60
40
20
0
Xlf-/- Ku80+/+
Xlf-/- Ku80+/-
Xlf-/- Ku80-/-
B
Xlf-/- Ku80+/+
Xlf-/- Ku80+/-
Xlf-/- Ku80-/-
Xlf-/- Ku80+/+
Xlf-/- Ku80+/-
Xlf-/- Ku80-/-
