# Supplementary material for: Synthetic lethality between PAXX and XLF in mammalian development
Source: Genes Dev. 2016 Oct 1;30(19):2152–7. doi: 10.1101/gad.290510.116 (PMC5088564; doi:10.1101/gad.290510.116)

Figure S2. ***Paxx*$^{-/-}$ mice are born at expected frequencies and show no overt phenotype.** A) Table showing the number of viable embryos transferred to recipient females and the number of viable mutant and non-mutant mice born. B) Representative picture of Paxx chimera (100%) as compared to a non-mutant control (males, 6 weeks of age). C) PCR gel electrophoresis of the 12 chimeras and the 3 recipient females. The four founders are clearly visible in the knock-out Paxx PCR. D) Mice were genotyped using ear snip biopsies and expected *vs.* observed numbers used to calculate chi-square. Bar graphs presenting selected genotype combinations are presented. E) Weight chart of male and female *Paxx*$^{-/-}$ mice as compared to *Paxx*$^{+/+}$ and *Paxx*$^{+/-}$ (*Paxx*$^{+}$) mice. No difference was observed between *Paxx*$^{+/+}$ and *Paxx*$^{+/-}$ mice.

A

Cas9	Embryo transferred	Recipients	Pups born	Coat colour	Sex	Paxx
Cas9$^{+/+}$	60	3	12	Albino 100%	Male (4)	chimera 100%
				Albino 100%	Female (7)	chimera 80%
				Albino 100%	Female (8)	chimera 80%
				Albino 100%	Female (15)	chimera 10%

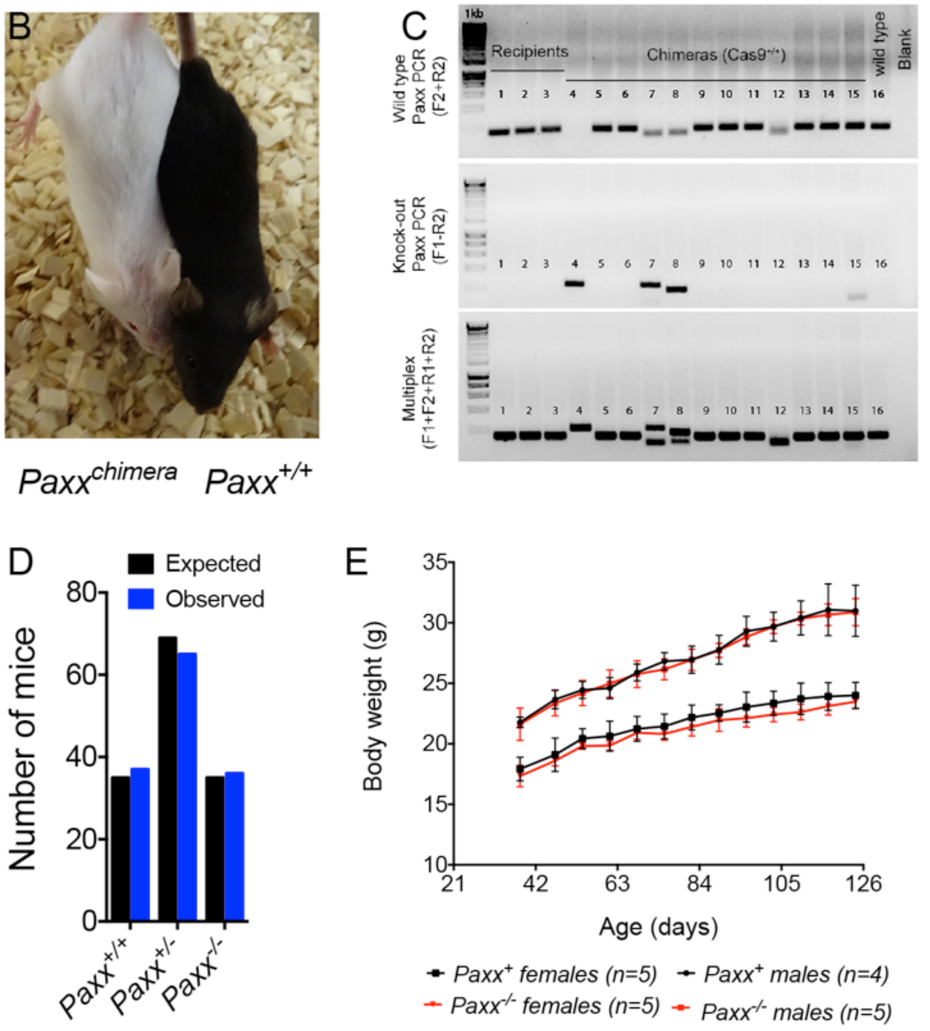

Supplement: Supplemental Material [file supp_30.19.2152_Supplemental_Fig_S2.pdf]
