# Supplementary material for: Synthetic lethality between PAXX and XLF in mammalian development
Source: Genes Dev. 2016 Oct 1;30(19):2152–7. doi: 10.1101/gad.290510.116 (PMC5088564; doi:10.1101/gad.290510.116)

Figure S9. *Paxx*$^{-/-}$*Xlf*$^{-/-}$ mouse embryonic fibroblasts show no synergistic increase in sensitivity to DSBs. A) Survival analysis of phleomycin sensitivity in primary MEFs of the indicated genotypes. One-way ANOVA (Dunnett's multiple comparisons test; ***$p<0.001$) statistical analysis was performed. B) Survival analysis of IR sensitivity in SV40-immortalized MEFs of the indicated genotypes. One-way ANOVA (Dunnett's multiple comparisons test; ***$p<0.001$) statistical analysis was performed. C) Bar graphs (mean ± SD) representing three independent (n=3) neutral comet experiments measuring DSB repair in WT, *Paxx*$^{-/-}$, *Xlf*$^{-/-}$ or *Paxx*$^{-/-}$ *Xlf*$^{-/-}$ MEFs. Cells were mock-treated, treated with 40 µg/ml phleomycin for 2 hours, or phleomycin-treated (D) and left to recover (R) for 2 hours. The normalized comet tails (R/D) were plotted. Statistical analysis was performed using One-way ANOVA (Dunnett's multiple comparisons test; ****$p<0.0001$).

Balmus_Supplemental_Fig_S9

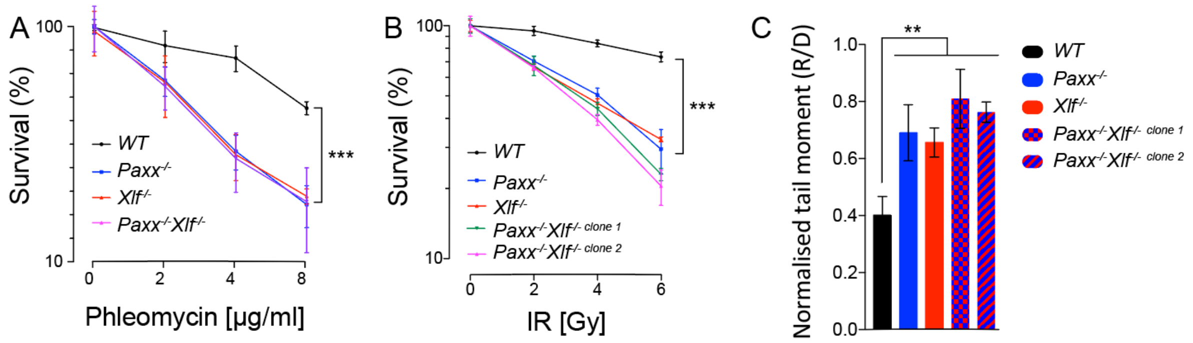

Supplement: Supplemental Material [file supp_30.19.2152_Supplemental_Fig_S9.pdf]
