# Supplementary material for: Synthetic lethality between PAXX and XLF in mammalian development
Source: Genes Dev. 2016 Oct 1;30(19):2152–7. doi: 10.1101/gad.290510.116 (PMC5088564; doi:10.1101/gad.290510.116)
Supplement: Supplemental Material [file supp_30.19.2152_Supplemental_Table_S3.pdf]

Supplemental Table S3. ***Paxx$^{-/-}$* *Xlf$^{-/-}$* embryos start dying after E14.5.**

Embryos were genotyped by PCR analysis of DNA extracted from the yolk sacks. Day of vaginal plug was considered E0.5. Expected values represent the combined Mendelian percentages and numbers from several crosses involving different parental genotypes of the *Paxx/Xlf* mice at E10.5 (A) and E18.5 (B). The significant values of observed and expected genotype frequencies were calculated using the $\chi 2$ and the representative data was graphed in Figure 3.

A **E14.5**

Genotype		% Expected	% Observed	# Expected	# Observed
Paxx $^{+/+}$	*Xlf* $^{+/+}$	0	0	0	0
	Xlf $^{+/-}$	12	3	4	1
	Xlf $^{-/-}$	16	13	5	4
Paxx $^{+/-}$	*Xlf* $^{+/+}$	0	0	0	0
	Xlf $^{+/-}$	12	17	4	5
	Xlf $^{-/-}$	38	43	11	13
Paxx $^{-/-}$	*Xlf* $^{+/+}$	0	0	0	0
	Xlf $^{+/-}$	6	7	2	2
	Xlf $^{-/-}$	16	17	5	5
**TOTAL**		**100**	**100**	**30**	**30**

B **E18.5**

Genotype		% Expected	% Observed	# Expected	# Observed
Paxx $^{-/-}$	*Xlf* $^{+/+}$	25	38	6	9
	Xlf $^{+/-}$	50	50	12	12
	Xlf $^{-/-}$	25	13	6	3(2)
**TOTAL**		**100**	**100**	**24**	**24**
