# Supplementary material for: Synthetic lethality between PAXX and XLF in mammalian development
Source: Genes Dev. 2016 Oct 1;30(19):2152–7. doi: 10.1101/gad.290510.116 (PMC5088564; doi:10.1101/gad.290510.116)

Figure S7. ***Paxx****$^{-/-}$* ***Xlf*** *$^{-/-}$* **embryos show increased genomic instability and apoptosis in the central nervous system.** A) Representative picture of E9.5 embryos of the selected genotypes. Scale bar 1 mm. B) Representative picture of a litter at E10.5. C) Representative images of cleaved caspase 3- and γH2AX-positive cells in neural tube of E10.5 embryos of the represented genotypes. Bar graphs (mean ± SD) present quantification of the % pan-nuclear γH2AX- and cleaved caspase 3-positive cells. More than 500 cells/embryo were counted (n≥3/genotype). One-way ANOVA (Dunnett's multiple comparisons test; *p<0.01; **p<0.001; ***p<0.001; ****p<0.0001) statistical analysis was performed. D) Representative images of cleaved caspase 3- and γH2AX-positive cells in neural tube of E14.5 embryos of the represented genotypes. Bar graphs (mean ± SD) present quantification of the % pan nuclear γH2AX- and cleaved caspase 3-positive cells. More than 1000 cells/embryo were counted (n≥3/genotype). Statistical analysis was performed using One-way ANOVA (Dunnett's multiple comparisons test; **p<0.001).

Balmus_Supplemental_Fig_S7

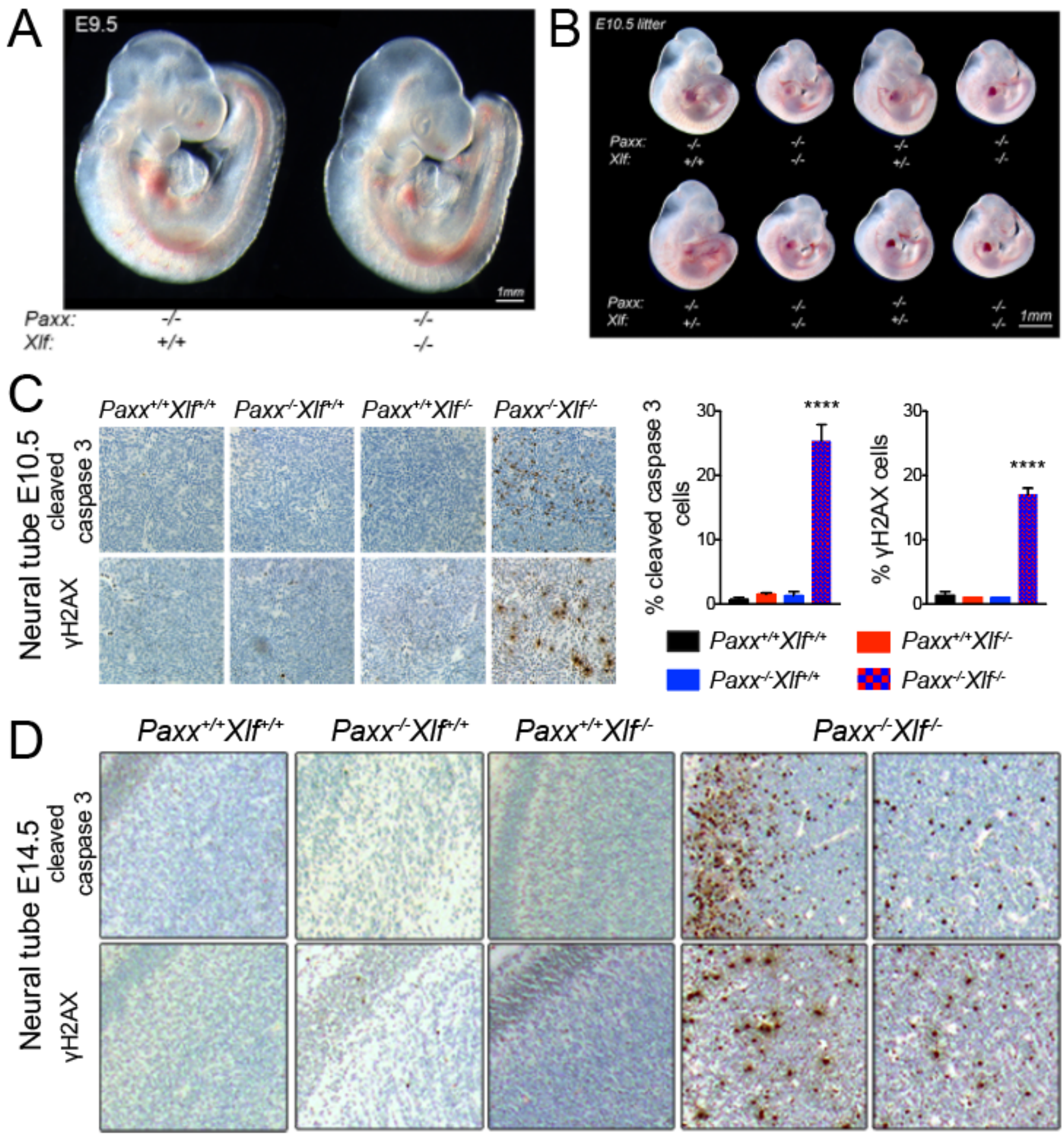

Supplement: Supplemental Material [file supp_30.19.2152_Supplemental_Fig_S7.pdf]
