# Supplementary material for: Synthetic lethality between PAXX and XLF in mammalian development
Source: Genes Dev. 2016 Oct 1;30(19):2152–7. doi: 10.1101/gad.290510.116 (PMC5088564; doi:10.1101/gad.290510.116)
Supplement: Supplemental Material [file supp_30.19.2152_Supplemental_Table_S1.pdf]

Supplemental Table S1. *Paxx*$^{-/-}$ mice do not show hematological abnormalities.

Complete blood counts of mice of the represented genotypes are presented. Mice were bled via tail bleed and complete blood counts were done. Whole blood (100-500 µl) was placed in 0.5-ml lithium heparinized gel separator tubes (Becton Dickinson). Each sample was analyzed within 2 hours of acquisition.

Genotype	*Paxx* $^{+/+}$				*Paxx* $^{-/-}$			
Age In Weeks	6							
Gender	Female							
White blood cells (WBC) x10^3/µl	3.6	6.89	6.01	5.37	6.53	5.45	3.81	3.94
Red blood cells (RBC) x10^6/µl	8.74	9.49	8.79	8.49	10.64	9.25	9.17	9.11
Hemogoblin (Hgb) g/dL	14.78	16.45	15.14	14.28	17.11	15.86	15.8	15.01
Hematocrit (HCT) %	41.08	43.65	42.19	39.9	47.88	43.48	43.1	41.91
Platlets (Plt) x10^3/µl	721	944	955	762	730	1020	985	909
Mean Corpuscular Volume (MCV) fl	47	46	48	47	45	47	47	46
Mean Corpuscular Hemoglobin (MCH) pg	16.91	17.33	17.22	16.82	16.08	17.15	17.23	16.48
Mean Corpuscular Hemoglobin Concentration (MCHC) g/dL	35.98	37.69	35.89	35.79	35.74	36.48	36.66	35.81
Red cell distribution width (RDW) %	12.96	13.26	12.02	13.45	12.59	13.05	12.81	13.33
Mean Platelet Volume (MPV) fl	5.3	5.66	4.76	5.55	5.09	5.17	5.32	5.13
Percent Lymphocytes	85.7	67.2	81	80.6	79.6	76.5	84.2	81.2
Percent Monocytes	2.7	5.6	3.6	4	4.2	4	3.9	4.1
Percent Granulocytes	11.6	27.2	15.4	15.4	16.2	19.5	11.9	14.7
Num Lymphocytes	3	4.6	4.8	4.3	5.1	4.1	3.2	3.2
Num Monocytes	0	0.3	0.2	0.2	0.2	0.2	0.1	0.1
Num Granulocytes	0.6	2	1	0.9	1.2	1.1	0.5	0.6

* mice were bled via tail bleed
