# Supplementary material for: Synthetic lethality between PAXX and XLF in mammalian development
Source: Genes Dev. 2016 Oct 1;30(19):2152–7. doi: 10.1101/gad.290510.116 (PMC5088564; doi:10.1101/gad.290510.116)

Figure S5. ***Paxx$^{-/-}$Atm$^{-/-}$* *mice show no further defect in lymphocyte numbers and CSR compared to Atm$^{-/-}$ mice.*** A) Total splenocytes, splenic B-cell and splenic T-cell numbers in WT, *Paxx*$^{-/-}$, *Atm*$^{-/-}$ and *Atm*$^{-/-}$ *Paxx*$^{-/-}$ mice. Bar graphs represent mean ± SD. *Atm*$^{-/-}$ and *Atm*$^{-/-}$ *Paxx*$^{-/-}$ mice show a significant reduction of total cell numbers as well as B and T cells in the spleen as compared to *Paxx*$^{-/-}$ mice (One-way ANOVA: Dunnett's multiple comparisons test; *p<0.01; **p<0.001; ***p<0.001; 6-8 weeks old, female mice), but no difference when *Atm*$^{-/-}$ is compared to *Atm*$^{-/-}$ *Paxx*$^{-/-}$ (splenocytes, p=0.84; splenic B-cells p=0.96; splenic T-cells p=0.94). B) Splenic B-cells were stimulated with α-CD40/IL-4 or LPS/IL-4 to switch to IgG1, and LPS to switch to IgG2b or IgG3. α-CD40-treated cells were used as negative controls. After 96 hours, cells were assayed for isotype switching by flow cytometry, with the percentage of IgG1+, IgG2b+ or IgG3+ B220+ B-cells indicated. Bar graphs represent mean ± SD. *Atm*$^{-/-}$ *Paxx*$^{-/-}$ mice show no significant reduction of %B220+ IgG1+, %B220+ IgG2+ and %B220+ IgG3+ cells in the spleen as compared to *Atm*$^{-/-}$ mice (One-way ANOVA: Dunnett's multiple comparisons test; 6-8 weeks old, female mice; %B220+ IgG1+ (p=0.99), %B220+ IgG2+ (p=0.99) and %B220+ IgG3+ (p=0.99)).

Balmus_Supplemental_Fig_S5

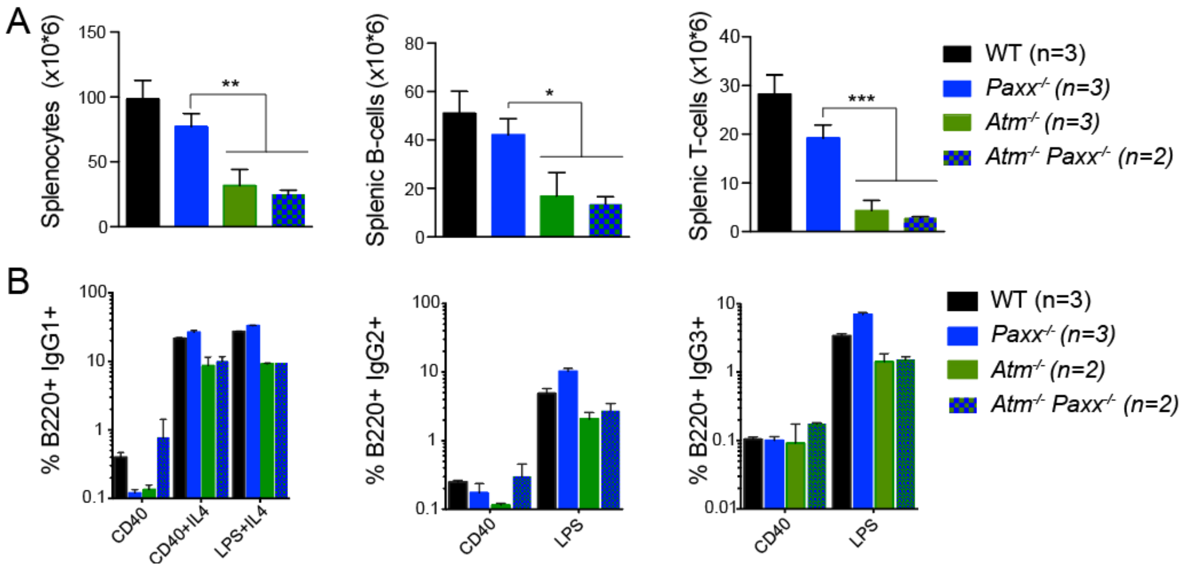

Supplement: Supplemental Material [file supp_30.19.2152_Supplemental_Fig_S5.pdf]
