# Supplementary material for: Synthetic lethality between PAXX and XLF in mammalian development
Source: Genes Dev. 2016 Oct 1;30(19):2152–7. doi: 10.1101/gad.290510.116 (PMC5088564; doi:10.1101/gad.290510.116)

Figure S3. *Paxx*$^{-/-}$ mice show no increased genomic instability and no overt immune deficiency. A) Micronucleus formation in *Paxx*$^{+/+}$ as compared to *Paxx*$^{-/-}$, *Xlf*$^{-/-}$ and *Atm*$^{-/-}$ mice. Cell counts from the thymus (B) and bone marrow (C) of *Paxx*$^{+/+}$ (n=4) as compared to *Paxx*$^{-/-}$ (n=4) littermate control mice. For bone marrow B cell development B cells were identified as B220+ and subsequently separated into pre-pro B cells (CD24$^{low/neg}$ and BP-1^{neg}) and developing B cells (CD24^{hi}). Developing B cells were classified into five subsets on the basis of surface IgM and IgD expression. There was a significant difference (Student's t-test; *$p<0.05$) between early and late mature B cells between *Paxx*$^{+/+}$ and *Paxx*$^{-/-}$ mice. D) ELISA assay for Ig, IgG1 and IgG2a titres upon immunisation with 100µg of purified fragment C of tetanus toxin. E) Splenic B-cells were stimulated with α-CD40/IL-4 or LPS/IL-4 to switch to IgG1, and LPS to switch to IgG2b or IgG3. α-CD40-treated cells were used as negative controls. After 96h, cells were assayed for isotype switching by flow cytometry, with the percentage of IgG1+, IgG2b+ or IgG3+ B220+ B-cells indicated.

Balmus_Supplemental_Fig_S3

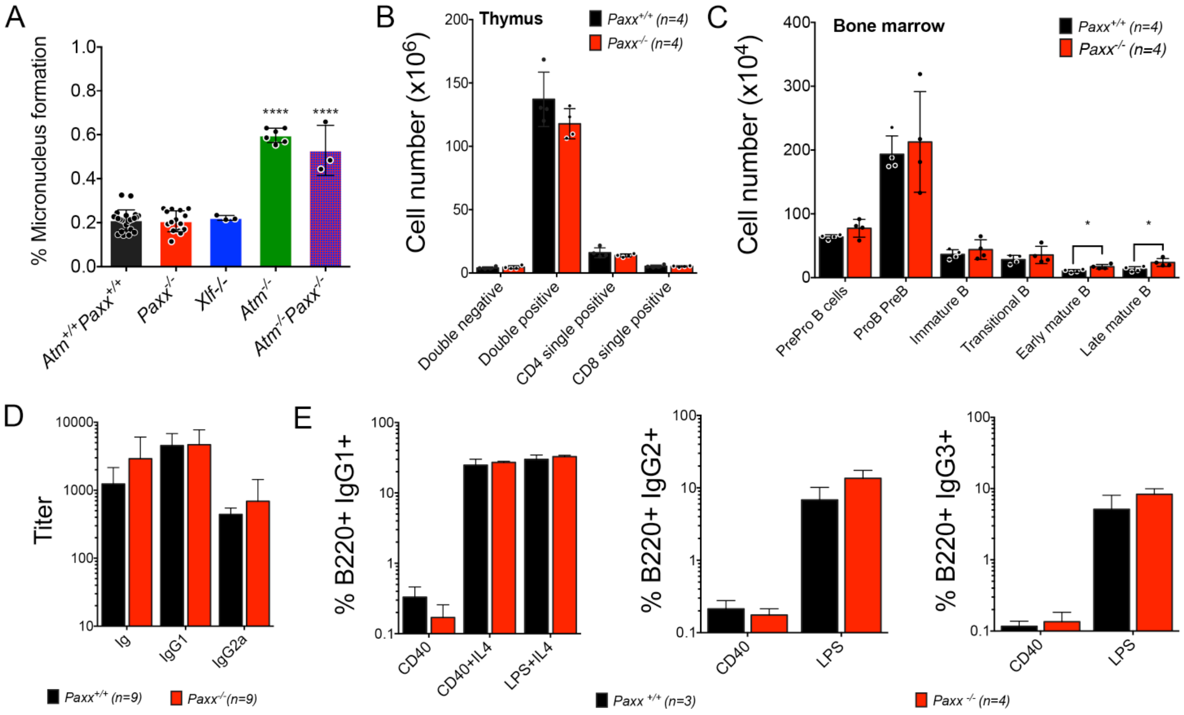

Supplement: Supplemental Material [file supp_30.19.2152_Supplemental_Fig_S3.pdf]
